# Lung cancer screening provider recommendation and completion in black and White patients with a smoking history in two healthcare systems: a survey study

**DOI:** 10.1186/s12875-024-02452-y

**Published:** 2024-06-07

**Authors:** Sandra J. Japuntich, Kristen Walaska, Elena Yuija Friedman, Brittany Balletto, Sarah Cameron, Joshua Ray Tanzer, Pearl Fang, Melissa A. Clark, Michael P. Carey, Joseph Fava, Andrew M. Busch, Christopher Breault, Rochelle Rosen

**Affiliations:** 1 Hennepin Healthcare, 730 South 8th St., Minneapolis, MN 55415 USA; 2 Hennepin Healthcare Research Institute, 701 Park Ave., PP7.700, Minneapolis, MN 55415 USA; 3 Department of Medicine, University of Minnesota Medical School, 401 East River Parkway, VCRC 1st Floor, Suite 131, Minneapolis, MN 55455 USA; 4 The Miriam Hospital, Coro Center West, 1 Hoppin St., Suite 309, Providence, RI 02903 USA; 5 Rhode Island Hospital, 130 Plain St., Providence, RI 02903 USA; 6 Brown University School of Public Health, One Davol Square, 121 South Main St, Providence, RI 02903 USA; 7 Department of Psychiatry and Human Behavior, Brown University, 75 Waterman St, Providence, RI 02912 USA

**Keywords:** Lung neoplasms, Early detection of cancer, Risk, Healthcare disparities

## Abstract

**Background:**

Annual lung cancer screening (LCS) with low dose CT reduces lung cancer mortality. LCS is underutilized. Black people who smoke tobacco have high risk of lung cancer but are less likely to be screened than are White people. This study reports provider recommendation and patient completion of LCS and colorectal cancer screening (CRCS) among patients by race to assess for utilization of LCS.

**Methods:**

3000 patients (oversampled for Black patients) across two healthcare systems (in Rhode Island and Minnesota) who had a chart documented age of 55 to 80 and a smoking history were invited to participate in a survey about cancer screening. Logistic regression analysis compared the rates of recommended and received cancer screenings.

**Results:**

1177 participants responded (42% response rate; 45% White, 39% Black). 24% of respondents were eligible for LCS based on USPSTF_2013_ criteria. One-third of patients eligible for LCS reported that a doctor had recommended screening, compared to 90% of patients reporting a doctor recommended CRCS. Of those recommended screening, 88% reported completing LCS vs. 83% who reported completion of a sigmoidoscopy/colonoscopy. Black patients were equally likely to receive LCS recommendations but less likely to complete LCS when referred compared to White patients. There was no difference in completion of CRCS between Black and White patients.

**Conclusions:**

Primary care providers rarely recommend lung cancer screening to patients with a smoking history. Systemic changes are needed to improve provider referral for LCS and to facilitate eligible Black people to complete LCS.

**Supplementary Information:**

The online version contains supplementary material available at 10.1186/s12875-024-02452-y.

## Background

Lung cancer screening (LCS) of high-risk individuals annually using Low Dose CT (LDCT) can reduce lung cancer mortality [1, 2]. A meta-analysis of eight trials found a 21–25% increase in lung cancer diagnosis and a 19% reduction in lung cancer mortality in the LCS groups versus the control groups as well as a number needed to screen of 250 to prevent 1 lung cancer death [3]. The United States Preventative Services Task Force (USPSTF) recommends annual LCS with LDCT for those at high risk for lung cancer. Initially, USPSTF guidelines reflected National Lung Screening Trial inclusion criteria (current and recent [quit < 15 years] former smoking, aged 55–80, with a 30-pack year smoking history [pack years = # of years smoking X packs smoked per day]). Updated USPSTF 2021 guidelines reduced the age to start LCS to 50 and the pack year requirement to 20 [4]. 

Despite evidence of life-saving benefit, LCS has been poorly utilized. In 2018, only 5% of those eligible (per USPSTF guidelines) had a LCS according to American College of Radiology LCS reports [5] and only 21% in 2019 using self-report data [6]. It is unclear what the rate limiting factor is for LCS: provider recommendations for the test, or patient completion of the test once recommended. An understanding what is undermining LCS utilization is needed in order to better tailor strategies to increase utilization.

It is particularly important for eligible Black people with a smoking history to receive LCS as they are diagnosed at later stage, Black men have the highest prevalence of lung cancer of any racial/ethnic group, and lung cancer is more likely to be fatal in Black men [7, 8]. If lung adenocarcinoma is caught in an early stage, lung cancer mortality is similar for Black and non-Black people [9]. LCS is more effective at reducing lung cancer mortality in Black people compared to White people [10, 11]. To reduce the disparities in lung cancer survival, Black people with a smoking history require similar or greater LCS uptake their White counterparts. Past findings have been mixed with regards to disparities in uptake of LCS with some finding similar uptake of LCS among Black and White survey respondents (although uptake, in general, was low) [12, 13], and some finding lower uptake in racial minority groups [14]. Utilization of other cancer screenings such as colorectal cancer screening is lower among Black people relative to White people [15]. Therefore, it is important to assess whether similar disparities develop in LCS as LCS is adopted more broadly. The updated guidelines may cause increases in the volume of screening that could exacerbate inequities in LCS. As a result, healthcare systems need to examine equity in screening utilization to ensure that this does not occur [16]. 

The current study used survey data from patients in two healthcare systems to investigate questions about LCS access and completion, namely: (1) Are those who meet USPSTF eligibility criteria for LCS being recommended for LCS by their providers? (2) Are those who are recommended LCS completing screening? (3) Is LCS underutilized compared to colorectal cancer screening (an imaging test recommended for a similar age range) [4]? (4) Are there differences in provider recommendation for LCS or CRC or patient completion of LCS or CRC by race? We compared lung and colorectal cancer screening because both are preventive tests for older adults. We hypothesized that (a) LCS recommendations and uptake would be lower than CRC screening; (b) Black patients would have lower reported provider recommendations and (c) screening test completion than White patients across LCS and CRC.

## Methods

### Data collection sites

This study had two recruitment sites, Lifespan Medical System in Rhode Island and Hennepin Healthcare in Minnesota. Lifespan is a large healthcare provider system including 5 hospitals. Hennepin Healthcare is an urban academic medical center serving Hennepin County. It has one hospital and several ambulatory care clinics.

### Participants

The study invited a total of 3,000 patients to participate (1,500 per healthcare system) who met the inclusion criteria. These individuals were randomly selected from the electronic medical record. To ensure a diverse representation, Black patients were intentionally oversampled, making up 50% of those invited to participate. The inclusion criteria were: having a medical visit within the past 12 months (to increase the likelihood of having accurate contact information), having a current or past history of cigarette smoking, being between the ages of 55 and 80, and residing in either Minnesota or Rhode Island. As the survey data collection began prior to the 2021 LCS guideline update, the eligibility criteria do not reflect the updated guideline, which lowered the age of eligibility to 50. The exclusion criteria included individuals who had opted out of all research participation, those with cognitive impairment, those without a valid mailing address, deceased individuals, and those who had a chart documented language preference other than English. Inclusion criteria/exclusion criteria were assessed via chart review. We confirmed age and cigarette smoking history on the survey. Individuals who were not aged 55–80 or who did not have a cigarette smoking history were excluded.

### Procedures

The electronic medical record was used to identify the sample; all data reported herein are from the survey. This was done for two reasons: first, linking medical record data to the survey would have made the survey identifiable and required written informed consent, and second, most primary care and CT scans for LCS for Lifespan patients are performed outside of the Lifespan health system. Data collection occurred from February 2021 through January 2022. Initially, a phone survey protocol was implemented to ensure participants understood the questions as intended (February-April 2021). Subsequently, a mailed survey option was introduced (March 2021-January 2022). In the phone survey protocol, potential participants received an invitation letter and a study fact sheet, providing information about the study and an opportunity to decline participation. Those who chose not to opt out were invited to complete the phone survey and were compensated with $10 upon completion. In the mixed mode protocol, participants were sent two mailings, spaced one month apart. The first mailing included an invitation letter with a phone number for opting out, a study fact sheet, a paper survey, a prepaid incentive of $10 (regardless of participation), and a postage-paid self-addressed envelope. The second mailing sent to people who did not respond or opt out was the same, except for the absence of the incentive. For those who didn’t opt out or respond by mail, researchers reached out via phone and offered options to complete the survey either over the phone or through mail.

### Measures

The full survey and source table can be found in the appendix. Measures relevant to the current study are described below.

*Demographics.* Participants reported their gender, age, race, ethnicity, insurance status, and educational attainment [17]. 

*Guideline eligibility calculation.* We computed LCS eligibility using the 2013 USPSTF guideline eligibility criteria (USPSTF_2013_). To confirm age, we asked “what is your current age?” [17] To confirm smoking history, we asked if they had ever smoked ≥ 100 cigarettes [17]. Those who reported having smoked for ≥ 30 pack years and had not been quit for ≥ 15 years were eligible (participants were asked if they now smoke every day, somedays or not at all [current/somedays = currently smoking, not at all = formerly smoking]) [17]. Finally, we asked the average number of cigarettes they smoked per day to assess if they had a ≥ 30 pack year history [2]. 

*Patient reported provider recommendation for LCS.* To assess for whether a provider had recommended LCS, participants were asked, “Thinking only of lung cancer, has a doctor ever recommended a CT (or CAT) scan to check for lung cancer?”

*Patient reported completion of LCS.* To assess for completion of LCS, participants were asked, “Have you ever had a CT (or CAT) scan to check for lung cancer?” [18].

*Patient reported provider recommendation for colorectal cancer screening (CRCS).* With regards to provider recommendations regarding CRCS, participants were asked whether a doctor had recommended a stool test, colonoscopy, or sigmoidoscopy [19]. 

*Patient reported completion of CRCS.* Participants were asked if they had ever received a stool test, sigmoidoscopy or colonoscopy [19]. 

### Data analysis

Analyses were conducted in R version 4.1.3. For individual variables, missing data were generally close to 5%, and no more than 10%. Multiple imputation was used, with modeled results representing the average of 100 imputed datasets. Thus, because of multiple imputation, we report estimates with 95% confidence intervals. There were no site differences in the results, so we report on the overall sample.

To assess whether the primary care provider’s recommendations for LCS were consistent with guidelines, we estimated patient-reported provider recommendations for LCS (based on USPSTF_2013_ guidelines; we do not report on the 2021 guidelines as these were not yet in practice when data collection began). To assess whether patients recommended for LCS were being screened, we estimated patient reported completion of LCS.

To compute comparisons between LCS and CRCS we estimated the number of patients who reported a provider recommendation for CRCS and, of those recommended, the number who reported completing CRCS. We then conducted a comparison of proportions based on the *F* and Chi-squared distributions for the patient-reported recommendations for LCS compared to the patient-reported recommendations for CRCS; the proportion of patients who reported a provider recommendation for LCS compared to those reporting having completed LCS; and proportion of patients who reported a provider recommendation for any CRCS test compared to those who reported having completed CRCS.

Finally, to test for differences in LCS completion amongst eligible Black vs. White patients we used logistic regression analysis. Patient-reported gender and insurance status were included in the logistic regression analysis. The selection of gender and insurance status was based on a preliminary cluster analysis of patient demographics, which identified gender and insurance status as distinguishing the largest differences between patients. Differences in terms of referrals and screen rates did not demonstrate meaningful differences between groups based on gender identity and insurance, so these will not be commented on in more detail. Reported results are estimates (aORs) from the regression model averaging across gender and insurance status groups. Raw regression output is not presented because it is not directly interpretable due to the many interaction terms.

## Results

**Fig. 1:**
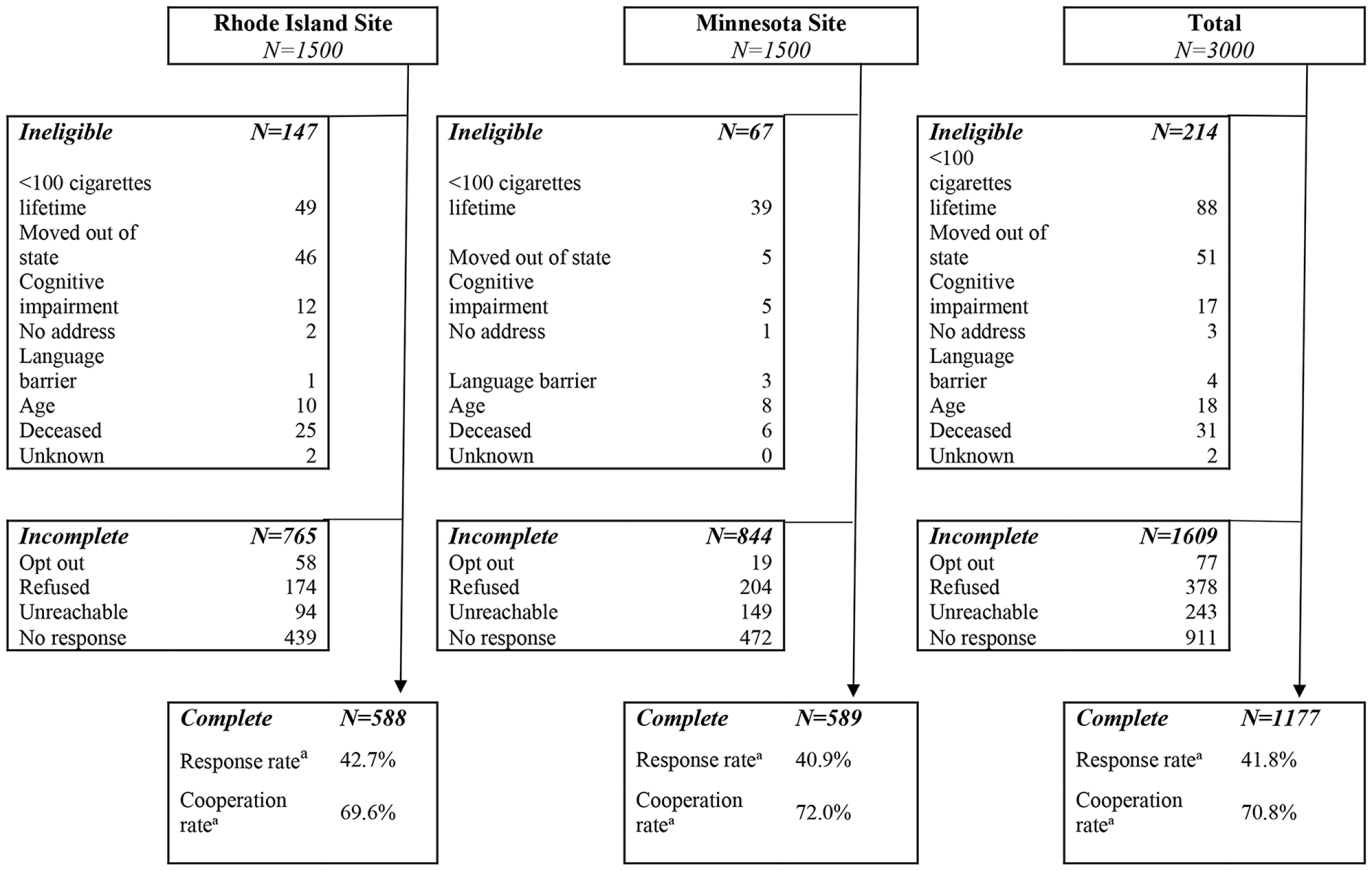
Study Flow by Site. ^a^Calculated using the AAPOR Outcome Rate Calculator version 4.1

The survey response rate was 42% and the cooperation rate (rate of response among people who had valid contact information) was 71% (see Fig. 1). Of the 3000 participants invited, 1177 participants completed the survey, 214 were ineligible, and 1609 did not complete the survey. 21% of the sample (250 patients) met USPSTF_2013_ eligibility criteria for LCS. See Table 1 for respondent demographics.

**Table 1:** Demographics of survey respondents (*N*=1177)

Demographic Category	Demographic Variable			
**Sociodemographic**	Age (*M*, *SD*)		66.1	6.7
	Female sex (n, %)		600	51.8%
	Education (n, %)	< High school graduate	161	14.2%
		High school/GED	335	28.5%
		Some college	368	32.4%
		College graduate	132	11.6%
		> College	141	12.4%
	Race (n, %)	White (non-Hisp)	534	46.7%
		Hispanic/Latino	39	3.4%
		Black (non-Hisp)	489	42.7%
		Asian/Pacific Islander	6	0.3%
		American Indian/Alaska Native	39	3.3%
		Other	39	3.4%
**Tobacco use history**	Combustible Cigarette use (n, %)	Current	462	39.6%
		Former	705	60.4%
	Cigarettes/day lifetime (*M*, *SD*)		14.3	10.0
	Years smoked (*M*, *SD*)		28.2	15.4
	Pack years (*M*, *SD*)		21.3	19.5
**Health history**	BMI (*M*, *SD*)		29.49	7.10
	COPD/Emphysema (n, %)		265	22.8%
	Cancer History (n, %)		304	26.1%
	Family lung cancer history (n, %)		229	19.5%
**Socioeconomic Status**	1 (low SES) – 10 (high SES) (*M*, *SD*)		5.38	3.34
**Insurance**	Are you covered by health insurance or some kind of other healthcare plan?	Yes	297	25.8%
		No	854	74.2%

Of those classified as eligible for LCS by USPSTF_2013_, 37% reported receiving a recommendation for LCS from their doctor. Of those who were eligible for screening based on USPSTF_2013_ guidelines and who reported being recommended for screening by their providers, 88% reported receiving LCS.

With regards to colorectal cancer screening, 44% of participants reported having a provider recommend a stool test using a home kit. Of these, 74% had received a stool test. Regarding procedural screening, 90% reported a provider recommended a sigmoidoscopy or colonoscopy. Of these, 83% reported having received a sigmoidoscopy or colonoscopy, with the majority of the tests (91%, 95% CI [89%, 94%]) being colonoscopies.

Participants were significantly more likely to have received guideline recommended colorectal cancer screening than LCS (OR = 7.63, 95% CI [5.29, 10.06]).

We then compared LCS and CRCS recommendations and completion by race (Black vs. White). Of those eligible for screening based on the USPSTF_2013_ guidelines, 35% of Black and 45% of White patients reported that their provider recommended LCS (Table 2). Of those who received a recommendation for LCS, 78% of Black patients and 97% of White patients reported receiving LCS. There were no differences between White and Black patients in provider recommendation or completion of colorectal cancer screening (Table 3).

**Table 2:** Lung cancer screening completion by guideline, risk status and race

Patient Race	Eligible (USPSTF_2013_)			Provider recommendations (of eligible)			Screens received (of referred)		
	Complete Cases	Estimate	95% CI	Complete Cases	Estimate	95% CI	Complete Cases	Estimate	95% CI
White	127 / 531	27.9%	24.7%,30.9%	63 / 121	45.1%	35.2%,56.1%	55 / 58	96.9%	79.0%,100.0%
Black	90 / 483	14.0%	10.3%,18.1%	53 / 85	35.3%	23.2%,49.5%	26 / 31	78.1%	38.0%,100.0%
All^	217 / 797	23.6%*	20.6%,26.2%	90 / 206	37.1%	30.3%,46.8%	81 / 89	88.1%^+^	82.5%,92.6%

US Preventive Services Task Force (USPSTF), 2013 indicated the 2013 guidelines for lung cancer screening ^All reported races; *Significant difference in eligibility for screening between Black and White patients *p* < .05; ^+^Significant difference in completion of screening between Black and White patients among those referred for screening *p* < .05; Complete Cases columns represents the raw frequencies for complete cases ignoring missing data. Frequencies will be slightly different when compared to Table 1 because of this

**Table 3:** Colorectal cancer screening completion by race

Test	Patient Race	Referrals			Screens received (of referred)		
		Complete Cases	Estimate	95% CI	Complete Cases	Estimate	95% CI
Stool test	White	311 / 523	41.8%	37.9%, 44.7%	161 / 211	73.9%	70.1%, 80.7%
	Black	258 / 480	44.9%	39.9%, 49.8%	131 / 184	75.5%	68.5%, 81.3%
	All	569 / 1003	43.6%	40.6%, 46.2%	292 / 395	74.4%	71.4%, 78.6%
Sigmoidoscopy / colonoscopy	White	466 / 527	92.1%	52.4%, 96.1%	426 / 466	92.0%	62.3%, 96.4%
	Black	385 / 482	85.8%	68.7%, 93.0%	344 / 383	88.1%	82.4%, 96.3%
	All	851 / 1009	89.8%	87.0%, 92.0%	770 / 849	83.1%	80.9%, 85.4%
Any colorectal cancer screening test	White	492 / 530	93.2%	90.7%, 95.3%	462 / 492	94.2%	92.1%, 96.2%
	Black	440 / 484	90.9%	86.7%, 93.8%	410 / 440	92.3%	89.1%, 95.4%
	All	932 / 1014	92.1%	89.9%, 93.9%	872 / 932	93.4%	91.7%, 95.2%

Complete Cases columns represents the raw frequencies for complete cases ignoring missing data. Frequencies will be slightly different when compared to Table 1 because of this

## Discussion

The current study investigated whether, across two healthcare systems: (1) Those who met USPSTF eligibility criteria for LCS were being recommended LCS by their providers, (2) Those being recommended LCS were receiving it, (3) Provider recommendation and patient completion of LCS compares to another cancer screening test for older individuals (CRCS) and (4) There were disparities in LCS or CRCS in Black people compared to White people. Overall, we found that providers recommended LCS to a minority of people meeting USPSTF guidelines in contrast to recommending CRCS to a majority of people; LCS completion among those referred was high, although completion of LCS was lower among Black people compared to White people for whom providers recommended LCS. This was in contrast to completion of CRCS, which did not differ by race.

Most patients reported that their providers did not recommend LCS. About one-third of respondents meeting USPSTF eligibility criteria for LCS recalled having had a primary care provider recommend it. LCS referral is complicated by the eligibility criteria and because is not recommended for people with a “health problem that substantially limits life expectancy” [20]. To be selected for LCS people need to be well enough to receive treatment for lung cancer. We did not evaluate these criteria. People with a smoking history often have other smoking-related comorbidities affecting life expectancy. A meta-analysis of eight LCS trials found a rate of overdiagnosis (diagnosis of lung cancer among people who are going to die of something else) of 20% [3]. Overdiagnosis may be of particular risk to Black people who, when diagnosed in early stage, have similar or lower lung cancer mortality than non-Hispanic White people but lower overall survival [9, 21]. Thus, the optimal rate of LCS is likely lower than 100% but greater than the rate reported in this sample. Providers have previously reported barriers to adoption of LCS across all patients such as logistical barriers (e.g., time, insurance coverage), lack of knowledge, and the high prevalence of nodules that need to be managed [22, 23]. 

Despite low frequency of provider recommendations for lung cancer screening, the vast majority of participants recommended for LCS received it. This could reflect high interest in LCS, providers referring patients who are most concerned about LCS, or recall bias such that those who received LCS are more likely to remember being recommended LCS.

Overall, we found that participant reported provider recommendation for LCS (37% of patients) was lower than provider recommendation for CRCS (90% of patients). Participation in CRCS has been augmented by provider performance measures [24]. Due to the performance measures, health systems often have care coordinators to increase screening rates. Similar health system changes are needed to improve LCS referrals for eligible patients. Potential interventions include financial incentives for providers to refer for screening, continuous quality improvement, clinician reminders, audit and feedback, and screening navigation [25]. 

Black participants recommended LCS were less likely to complete it than White participants. In CRCS, most participants completed the test when recommended by their provider. Further, there were no racial differences in completion of CRCS among those recommended screening. The disparity in LCS completion is consistent with other studies that have found lower LCS completion after referral, lower completion of follow-up tests, repeat annual screens, and lung cancer treatment among Black people relative to White people [26–29]. Barriers to LCS for Black people include provider factors (e.g., mistrust of providers, lack of clear physician communication), recipient factors (e.g., incomplete understanding about the purpose of screening, stigma), and structural barriers (e.g., low access to care [convenience and cost], poverty, low education level, lack of insurance, and lack of a consistent primary care provider) [30–32]. 

LCS programs as well as their referral sources should deploy strategies to improve follow-through. High quality shared decision making targeting recipient concerns could improve uptake [33–35]. Navigators or community health workers can mitigate structural barriers leading to racial disparities in LCS completion [16, 36, 37]. Evidence from other cancer screenings suggest consistent provider recommendations, population management, and text or patient portal reminders can increase follow through [38]. Finally, point of care screening may reduce barriers to making a return visit.

Interpretation of study findings should consider study limitations. This was a single timepoint survey conducted among patients in two healthcare systems and may not reflect population level screening behavior. However, our results are consistent with previous research using population samples [5, 6]. We were not able to assess whether participants were healthy enough for LCS which might explain why some were not recommended LCS. Finally, this study used self-report and was not able to validate survey responses with chart data. Participants may not have remembered if they were referred and/or screened or been able to distinguish diagnostic tests from LCS. Previous research has found people have difficulty identifying whether they have had LCS [39]. 

## Conclusions

This study found that among patients established in a healthcare system, LCS remains underutilized. Providers are likely missing opportunities to refer high risk patients for screening. More research is needed to identify ways to improve provider recommendation of LCS including qualitative studies regarding provider reasons for not making recommendations for LCS. This study suggests disparities in LCS utilization between Black and White people are due, in part, to lower LCS completion among Black people. Future work should explore structural barriers to completion and develop care navigation interventions to support LCS.

### Electronic supplementary material

Below is the link to the electronic supplementary material.

Supplementary Material 1

## Data Availability

Data is available by contacting the corresponding author.

## Abbreviations

LCS: Lung cancer screening
USPSTF: United States Preventative Services Task Force
CRCS: Colorectal cancer screening
