# Supplementary material for: Lung cancer screening provider recommendation and completion in black and White patients with a smoking history in two healthcare systems: a survey study
Source: BMC Prim Care. 2024 Jun 7;25:202. doi: 10.1186/s12875-024-02452-y (PMC11157907; doi:10.1186/s12875-024-02452-y)
Supplement: Supplementary file 1 — Supplementary Material 1 [file 12875_2024_2452_MOESM1_ESM.docx]

Appendix: Survey source table

| Item number | Survey item | Response options | Source |
| --- | --- | --- | --- |
| 1 | Have you smoked at least 100 cigarettes in your entire life | Yes; No | Behavioral Risk Factor Surveillance System (2018 survey)[1] |
| 2 | Do you now smoke cigarettes every day, some days, or not at all? | Every day; Some days; Not at all | Behavioral Risk Factor Surveillance System (2018 survey)[1] |
| 3 | How long has it been since you quit smoking cigarettes? | Less than 1 year; 1 year to less than 5 years; 5 years to less than 10 years; 10 years to less than 15 years; 15 or more years | National Health and Nutrition Examination Survey (2017-2018)[2] |
| 4 | For how many years have you smoked? | ______ years | Adapted from shouldiscreen.com[3] |
| 5 | Considering all the years that you smoked, on average, how many cigarettes did you smoke per day (Note: there are 20 cigarettes in a pack) | ______cigarettes | Adapted from shouldiscreen.com[3] |
| 6 | Have any of your close relatives (mother, father, children, brothers, sisters, half-brothers, half-sisters) been diagnosed with lung cancer? | Yes; No; Don’t know/Not sure | Adapted from the Prostate, Lung, Colorectal and Ovarian Cancer Trial[4] |
| 7 | Has a doctor ever told you that you have any of the following conditions? (select all that apply) | Emphysema; Chronic Bronchitis; Chronic Obstructive Pulmonary Disease; None of the above | The Prostate, Lung, Colorectal and Ovarian Cancer Trial[4] |
| 8 | Have you ever been diagnosed with lung cancer? | Yes; No; Don’t know/not sure | Adapted from the Prostate, Lung, Colorectal and Ovarian Cancer Trial[4] |
| 9 | Have you received any of the following treatments for lung cancer? (select all that apply) | Surgery; Radiation Therapy; Thermal Ablation (freezing or burning of lung cancer using a needle); chemotherapy; none of the above | Created by study team |
| 10 | Have you been diagnosed with any type of cancer other than lung cancer? | Yes; No; Don’t Know/ Not Sure | Adapted from the Prostate, Lung, Colorectal and Ovarian Cancer Trial[4] |
| 11 | The next question is about CT (or CAT) scans. During this test you lie flat on your back on a table. While you hold your breath, the table moves through a donut shaped x-ray machine while the scan is done. Has your doctor ever recommended a CT (or CAT) scan for you? | Yes; No; Don’t know/not sure | Created by study team, definition of CT from Behavioral Risk Factor Surveillance System (2018 survey)[1] |
| 12 | Thinking only of lung cancer, has a doctor ever recommended a CT (or CAT) scan to check for lung cancer? | Yes; No; Don’t know/not sure | Created by study team |
| 13 | Have you ever had a CT (or CAT) scan to check for lung cancer? | Yes; No; Don’t know/not sure | Adapted from Delmerico et al. 2015[5] |
| 14 | When did you have your first CT scan to check for lung cancer? | Less than 6 months ago; 6 months to less than 1 year ago; 1 year to less than 2 years ago; 2 years to less than 3 years ago; 3 years to less than 5 years ago; more than 5 years ago | Created by study team |
| 15 | How many times have you had a CT scan to check for lung cancer? | 1; 2; 3 or more | Created by study team |
| 16 | When did you have your most recent scan to check for lung cancer? | Less than 6 months ago; 6 months to less than 1 year ago; 1 year to less than 2 years ago; 2 years to less than 3 years ago; 3 years to less than 5 years ago; more than 5 years ago | Created by study team |
| 17 | To what extent did the following happen because of a CT scan to check for lung cancer? (please select a response for each item) |  | Created by study team |
|  | The CT scan procedure made you anxious. | Very much; Somewhat; Not at all |  |
|  | The result of the CT scan made you anxious | Very much; Somewhat; Not at all |  |
|  | The CT scan caused you physical discomfort | Very much; Somewhat; Not at all |  |
| 18 | Because of your CT scan to check for lung cancer, did you have any of the follow-up tests listed below? (select all that apply) | A repeat CT scan in less than one year; A PET scan (where radioactive sugar is injected into your arm); A lung biopsy; Lung surgery; Other (please specify); did not have any follow-up tests | Created by study team |
| 19 | To what extent did any of the following happen because of the follow-up tests you had after your CT scan to check for lung cancer? (Please select a response for each item) |  | Created by study team |
|  | The follow-up tests caused you physical discomfort | Very much; Somewhat; Not at all |  |
|  | The follow-up tests caused you pain | Very much; Somewhat; Not at all |  |
|  | The follow-up tests caused you physical injury or harm | Very much; Somewhat; Not at all |  |
| 20 | Were you scheduled to have a CT scan to check for lung cancer but it was canceled or postponed because of the COVID-19 pandemic? | Yes; No; Don’t know/not sure | Created by study team |
| 21 | Do you plan to have a CT scan to check for lung cancer in the next 12 months? | Yes; No; Don’t know/not sure | Adapted from Delmerico et al. 2015[5] |
| 22 | Would you have a CT scan to check for lung cancer in the future if a doctor recommends it? | Yes; No; Maybe | Adapted from Delmerico et al. 2015[5] |
| 23 | In making decisions about whether to have a CT scan to check for lung cancer, how important are each of the following to you? (please select a response for each item) |  | Adapted from Caltaldo et atl 2016[6] |
|  | The location of the facility where you would get the test | Very important; Moderately important; Slightly important; Not important |  |
|  | The hours of operation of the facility where you would get the test | Very important; Moderately important; Slightly important; Not important |  |
|  | Your risk of lung cancer | Very important; Moderately important; Slightly important; Not important |  |
|  | The accuracy of the test | Very important; Moderately important; Slightly important; Not important |  |
|  | The cost of the test | Very important; Moderately important; Slightly important; Not important |  |
|  | Risk of being infected with COVID-19 | Very important; Moderately important; Slightly important; Not important |  |
| 24 | Below is a list of reasons why some people may decide to get a CT scan to check for lung cancer. Please select the reasons why you may decide to get this test in the future. (Select all that apply) | You want to be able to diagnose lung cancer as early as possible; You believe your health insurance will cover most of the cost; You want to follow doctors’ recommendations; You feel you are at risk for lung cancer; You are sick or have had medical problems (such as a cancer diagnosis; a cough; pain; difficulty breathing or weight loss);Other reason please specify; none of the above/you would not get a CT scan to check for lung cancer | Adapted from Delmerico et al. 2015[5] |
| 25 | A stool test is a test that may use a special kit at home to determine whether the stool contains blood. Did a doctor recommend this test for you? | Yes; No | Behavioral Risk Factor Surveillance System (2018 survey)[1] |
| 26 | Have you ever had a stool test using a home kit? | Yes; No | Behavioral Risk Factor Surveillance System (2018 survey)[1] |
| 27 | How long has it been since you had your last stool test using a home kit? | Less than 1 year ago; 1 year to less than 2 years ago; 2 years to less than 3 years ago; years to less than 5 years ago; 5 or more years ago | Behavioral Risk Factor Surveillance System (2018 survey)[1] |
| 28 | Sigmoidoscopy and colonoscopy are exams in which a tube is inserted in the rectum to view the colon for signs of cancer or other health problems. Did a doctor recommend either of these exams for you? | Yes; No | Behavioral Risk Factor Surveillance System (2018 survey)[1] |
| 29 | Have you ever had a sigmoidoscopy or colonoscopy? | Yes; No | Behavioral Risk Factor Surveillance System (2018 survey)[1] |
| 30 | For a sigmoidoscopy, a flexible tube is inserted into the rectum to look for problems. A colonoscopy is similar, but uses a longer tube, and you are usually given medication through a needle in your arm to make you sleepy and told to have someone else drive you home after the test. Was your most recent exam a sigmoidoscopy or a colonoscopy? | Sigmoidoscopy; Colonoscopy | Behavioral Risk Factor Surveillance System (2018 survey)[1] |
| 31 | How long has it been since you had your most recent sigmoidoscopy or colonoscopy? | Less than 1 year ago; 1 year to less than 2 years ago; 2 years to less than 3 years ago; 3 years to less than 5 years ago; 5 years to less than 10 years ago; 10 or more years ago | Behavioral Risk Factor Surveillance System (2018 survey)[1] |
| 32 | Were you scheduled to have a sigmoidoscopy or colonoscopy but it was canceled or postponed because of the COVID-19 pandemic? | Yes; No | Created by study team |
| 33 | There are many types of health insurance, such as coverage through an employer, coverage purchased directly, as well as government programs, like Medicare, Medicaid, and Tricare. Are you covered by any type of health insurance? | Yes; No; Don’t know/Not sure | National Health and Nutrition Examination Survey (2017-2018)[2] |
| 34 | Which of the following types of health insurance are you covered by? (select all that apply) | A plan purchased through and employer or union (including plans purchased through another person’s employer); A plan that you or another family member buys directly; Medicare; Medicaid or another state program; Tricare; Other (please specify) | Behavioral Risk Factor Surveillance System (2014 survey)[7] |
| 35 | Some insurance plans have an amount that you must pay for covered health care services before the insurance plan starts to pay. This is called a deductible. Does your insurance plan have a deductible? | Yes; No; Don’t Know/Not sure | Kaiser Family Foundation[8] |
| 36 | In general, how easy or difficult is it for you to afford to pay the deductible for your health insurance? | Very easy; Somewhat easy; Somewhat difficult; Very difficult | Kaiser Family Foundation[9] |
| 37 | What is your gender? | Male; Female; Transgender – Male to Female; Transgender Female to Male; Other (please specify) | Created by study team |
| 38 | What is your age? | _______years | Behavioral Risk Factor Surveillance System (2018 survey)[10] |
| 39 | Are you of Hispanic or Latino origin or descent? | Yes, Hispanic or Latino; No, not Hispanic or Latino | Adapted form the Prostate, Lung, Colorectal and Ovarian Cancer Trial[4] |
| 40 | What is your race (select all that apply) | White; Black or African American; Asian; Native Hawaiian or Other Pacific Islander; American Indian or Alaska Native; Other | Adapted from the Prostate, Lung, Colorectal and Ovarian Cancer Trial[4] |
| 41 | What is the highest grade or level of school that you have completed? | 8^th^ grade or less; some high school, but did not graduate; High school graduate or GED; Some college or 2-year degree; 4-year college graduate; More than 4-year college degree | Adapted from the Prostate, Lung, Colorectal and Ovarian Cancer Trial[4] |
| 42 | Without giving exact dollars, how would you describe your household’s financial situation right now? | Not enough money to pay some bills no matter how hard you try; Enough money to play bills, but have had to cut back; Enough money to pay bills without cutting back, but no “extras”; Enough money for “extras” | Krieger (1990)[11] |
| 43 | What is your current weight? | _________ pounds | Adapted from the Prostate, Lung, Colorectal and Ovarian Cancer Trial[4] |
| 44 | How tall are you (record your height in feet and inches) | ______feet & _____inches | Adapted from the Prostate, Lung, Colorectal and Ovarian Cancer Trial[4] |
| 45 | In general, how would your rate your overall health? | Excellent, Very good; Good; Fair; Poor | Behavioral Risk Factor Surveillance System (2018 survey)[10] |
| 46 | Do you have one ore more people whom you think of as your personal doctor or health care provider? | Yes; No | Behavioral Risk Factor Surveillance System (2014 survey)[7] |
| 47 | About how long has it been since you last visited a doctor for a routine checkup? A routine checkup is a general physical exam, not an exam for a specific injury, illness, or condition. | Less than 1 year ago; 1 year to less than 2 years ago; 2 years to less than 5 years ago; 5 or more years ago; Never | Behavioral Risk Factor Surveillance System (2014 survey)[7] |
| 48 | We would like to learn some of your feelings about your health and life in general. For each of these statements, please answer whether you strongly agree, agree, disagree, or strongly disagree. |  | Jacobson 1999[12] |
|  | When bad things happen, we are not supposed to know why, we are just supposed to accept them | Strongly agree; Agree; Disagree; Strongly disagree |  |
|  | People die when it is their time to die and nothing can change that | Strongly agree; Agree; Disagree; Strongly disagree |  |
|  | Everything that happens is a part of God’s plan | Strongly agree; Agree; Disagree; Strongly disagree |  |
|  | If bad things happen, it is because they were meant to be | Strongly agree; Agree; Disagree; Strongly disagree |  |
| 49 | Have you ever been discriminated against because of…? Please select a response for each item |  | Krieger (1990)[11] |
|  | Your age | Yes; No |  |
|  | Your race or ethnicity | Yes; No |  |
|  | Your gender | Yes; No |  |
|  | Your sexual orientation | Yes; No |  |
|  | Your appearance | Yes; No |  |
|  | Your income level | Yes; No |  |
| 50 | How often do each of the following things happen to you in medical settings? Please select a response for each item |  | Discrimination in Medical Settings[13] |
|  | You are treated with less courtesy than other people | Always; Most of the time; Sometimes; Rarely; Never |  |
|  | You are treated with less respect than other people | Always; Most of the time; Sometimes; Rarely; Never |  |
|  | You receive poorer service than others | Always; Most of the time; Sometimes; Rarely; Never |  |
|  | A doctor or nurse acts as if he or she thinks you are not smart | Always; Most of the time; Sometimes; Rarely; Never |  |
|  | A doctor or nurse acts as if he or she is afraid of you | Always; Most of the time; Sometimes; Rarely; Never |  |
|  | A doctor or nurse acts as if he or she is better than you | Always; Most of the time; Sometimes; Rarely; Never |  |
|  | You feel like a doctor or nurse is not listening to what you are saying | Always; Most of the time; Sometimes; Rarely; Never |  |

References (Appendix)

1. CDC. 2018 BRFSS questionnaire. 2023. https://www.cdc.gov/brfss/questionnaires/pdf-ques/2018_BRFSS_English_Questionnaire-508.pdf. Accessed 1 Aug 2023.

2. National Center for Health Statistics. NHANES 2017-2018 Overview. https://wwwn.cdc.gov/nchs/nhanes/continuousnhanes/overview.aspx?BeginYear=2017. Accessed 1 Aug 2023.

3. Lau YK, Caverly TJ, Cao P, Cherng ST, West M, Gaber C, et al. Evaluation of a personalized, web-based decision aid for lung cancer screening. Am J Prev Med. 2015;49:e125-129.

4. National Cancer Institute. PLCO questionnaires datasets.

5. Delmerico J, Hyland A, Celestino P, Reid M, Cummings KM. Patient willingness and barriers to receiving a CT scan for lung cancer screening. Lung Cancer Amst Neth. 2014;84:307–9.

6. Cataldo JK. High-risk older smokers’ perceptions, attitudes, and beliefs about lung cancer screening. Cancer Med. 2016;5:753–9.

7. CDC. 2014 BRFSS questionnaire. 2014. https://www.cdc.gov/brfss/questionnaires/pdf-ques/2014_BRFSS.pdf.

8. Kirzinger A, Sugarman E, Brodie M. Kaiser Health Tracking Poll: Future Directions for the ACA and Medicaid. Kaiser Family Foundation; 2017.

9. Poll: most Americans don’t realize how dramatically the Medicare-for-all proposals would eevamp the nation’s health care system. KFF. 2019. https://www.kff.org/health-reform/press-release/poll-most-americans-dont-realize-how-dramatically-medicare-for-all-proposals-would-revamp-nations-health-care-system/. Accessed 1 Aug 2023.

10. CDC. 2018 survey data. BRFSS. 2022. https://www.cdc.gov/brfss/annual_data/annual_2018.html. Accessed 1 Aug 2023.

11. Krieger N. Racial and gender discrimination: risk factors for high blood pressure? Soc Sci Med 1982. 1990;30:1273–81.

12. Jacobson CK. Denominational and racial and ethnic differences in fatalism. Rev Relig Res. 1999;41:9.

13. Peek ME, Nunez-Smith M, Drum M, Lewis TT. Adapting the everyday discrimination scale to medical settings: reliability and validity testing in a sample of African American patients. Ethn Dis. 2011;21:502–9.
